# THE USE OF ULTRASONOGRAPHY TO EVALUATE MUSCLE THICKNESS AND SUBCUTANEOUS FAT IN CHILDREN AND ADOLESCENTS WITH CYSTIC FIBROSIS

**DOI:** 10.1590/1984-0462/;2018;36;4;00015

**Published:** 2018

**Authors:** Rodrigo Pereira de Souza, Márcio Vinícius Fagundes Donadio, João Paulo Heinzmann-Filho, Rafael Reimann Baptista, Leonardo Araújo Pinto, Matias Epifanio, Paulo José Cauduro Marostica

**Affiliations:** a Programa de Pós-graduação em Saúde da Criança e do Adolescente, Universidade Federal do Rio Grande do Sul, Porto Alegre, RS, Brasil.; b Pontifícia Universidade Católica do Rio Grande do Sul, Porto Alegre, RS, Brasil.

**Keywords:** Ultrasonography, Body composition, Skinfold thickness, Cystic fibrosis, Pediatrics, Ultrassonografia, Composição corporal, Pregas cutâneas, Fibrose cística, Pediatria

## Abstract

**Objective:**

To compare muscle thickness and subcutaneous fat in cystic fibrosis (CF) patients and healthy controls using ultrasonography (US), and to correlate US findings with nutritional, clinical and functional variables.

**Methods:**

Patients aged 6 to 18 years old with a diagnosis of CF and healthy controls were included. Participants underwent anthropometric measurements, an ultrasonographic evaluation of muscle thickness and subcutaneous fat in the triceps, quadriceps, and gastrocnemius regions, and skinfold thickness measurements. Body fat percentage was estimated using skinfold measurement. Subjects with CF also underwent a pulmonary function assessment using spirometry.

**Results:**

We studied 39 CF patients and 45 controls. Alower body mass index was observed in CF patients (p=0.011). Body composition and muscle thickness were similar between the groups. Only calf (p=0.023) circumference and femur diameter (p<0.001) were lower in CF patients. Although there were no significant between-group differences in the comparison of US measurements of subcutaneous fat, CF patients exhibited decreased skinfold thickness in the triceps (p=0.031) and quadriceps (p=0.019). Moreover, there were weak and moderate correlations of US quadricep thickness with forced vital capacity (FVC) and lean mass, respectively. Moderate correlations of the triceps, quadriceps and gastrocnemius between US subcutaneous fat and skinfold measurements were found.

**Conclusions:**

Patients with CF presented a reduction in subcutaneous fat content. Muscle thickness correlated with FVC and nutritional parameters. In addition, US findings correlated positively with skinfold measurements.

## RESUMO

**Objetivo:**

Comparar a espessura muscular e a gordura subcutânea entre pacientes com fibrose cística (FC) e controles saudáveis e correlacionar os achados ultrassonográficos com variáveis nutricionais, clínicas e de função pulmonar.

**Métodos:**

Foram incluídos sujeitos (6 a 18 anos) com o diagnóstico de FC e indivíduos saudáveis. Foram realizadas medidas antropométricas, avaliação ultrassonográfica da espessura muscular e da gordura subcutânea do tríceps, quadríceps e da região do gastrocnêmio, além da quantificação das pregas cutâneas. O percentual de gordura corporal foi estimado pelas pregas cutâneas. Osindivíduos com FC também tiveram a função pulmonar avaliada por espirometria.

**Resultados:**

Foram incluídos 39 pacientes com FC e 45 controles. Ossujeitos com FC apresentaram do índice de massa corporal menor (p=0,011). Acomposição corporal e a espessura muscular foram similares entre os grupos. Apenas a circunferência da panturrilha (p=0,023) e o diâmetro do fêmur (p<0,001) foram menores nos pacientes com FC. Embora sem diferença na comparação dos achados ultrassonográficos da gordura subcutânea, os pacientes com FC apresentaram redução das dobras cutâneas do tríceps (p=0,0031) e do quadríceps (p=0,019). Além disso, observaram-se correlações fracas e moderadas da espessura do quadríceps pelo ultrassom com a capacidade vital forçada (CVF) e massa magra, respectivamente. Também houve correlações moderadas das pregas cutâneas do tríceps, quadríceps e gastrocnêmio com a gordura subcutânea avaliada pela ultrassonografia.

**Conclusões:**

Pacientes com FC apresentaram menor espessura da gordura subcutânea. A espessura muscular se correlacionou com a CVF e os parâmetros nutricionais, e a ultrassonografia apresentou correlação positiva com as pregas cutâneas.

## INTRODUCTION

Nutritional and respiratory impairment, well-known features of cystic fibrosis (CF), affect muscle strength, limiting exercise capacity and the ability to perform everyday activities, which are closely related to the quality of life in CF.^1^ ^,^ ^2^ ^,^ ^3^ ^,^ ^4^ Maintenance of adequate nutritional status plays an essential role in supporting the integrity of the respiratory system in this patient population, and better nutrition is associated with improved pulmonary function outcomes and fewer *Pseudomonas* infections in children with CF.^5^ Also, CF patients with a better nutritional status have a linear increase in growth and appear to have greater lung function benefits from weight gain interventions.^6^ Thus, a quantitative muscle and body fat content assessment might provide important information on clinical status.

Several methods are available to assess body composition, including a skinfold thickness measurement, bioelectrical impedance and ultrasonography(US).^6^ UShas been widely employed as a diagnostic method and a therapeutic adjunct, and is increasingly available in inpatient and outpatient settings. Additional advantages of US are its relatively low cost, lack of discomfort,^7^ and the fact that it can be used at the bedside even in patients with restricted mobility. In addition, a previous study has shown that quantitative muscle US can be adequately performed by non-healthcare professionals following minimal training periods.^8^ However, there is still little research into its use as a nutritional diagnosis in children and adolescents, who are healthy or sick. Although ultrasonography accuracy has not been evaluated for CF patients, the method has been shown to be sensitive and specific to detect abnormalities in patients with neuromuscular diseases.^9^

Muscle strength is proportional to the physiological cross-sectional area of the muscle, and can be effectively estimated by muscle thickness.^10^ Starkey etal.^11^ have used US to measure the effects of 14 weeks of resistance training on muscle thickness and observed small but significant increases in the right thigh muscle. Because the changes produced by treatment may be small, especially in the case of chronically ill patients with less potential for building muscle, the method used to measure muscle thickness must be highly sensitive to allow for the detection of slight changes.

Considering that US assessment of body composition in CF patients has not been explored in the literature, the objective of the present study was to compare muscle thickness and subcutaneous fat in children and adolescents with CF and healthy controls using US, as well as to correlate these US findings with nutritional, clinical, and functional variables in CF patients. Also, we sought to evaluate the subcutaneous fat measurement based on US as an alternative method compared to the conventional skinfold measurement in patients with CF and healthy controls.

## METHOD

A controlled cross-sectional study was carried out with children and adolescents with CF seen at the Pediatric Pulmonary Division of a university hospital. Healthy controls were recruited from a recreational/cultural program for school children at the same university. Both CF patients and healthy controls were consecutively recruited as approaching was made available. ACF diagnosis was based on two positive sweat tests (chloride concentration >60mEq/L) or a mutation analysis (the presence of two disease causing mutations).

In both groups, children and adolescents between 6 and 18 years of age were eligible for participation in the study. Healthy controls were recruited after answering a respiratory health questionnaire to rule out the presence of chronic diseases. Additional exclusion criteria included signs of pulmonary exacerbation in the previous two weeks in CF patients and not being capable of correctly performing the study procedures. Data were collected between November, 2013 and November, 2014.

Data on medical history were collected in the form of an interview during office visits. At first, signatures were obtained on consent and assent forms. For each patient, we collected anthropometric and demographic data (age and gender), and information on genetic mutation (if available), pancreatic insufficiency, clinical severity score (Shwachman-Kulczyck) and on chronic infection with *Pseudomonas. aeruginosa*. Chronic *Pseudomonas aeruginosa* infection was defined as a persistent colonization for at least six consecutive months (three consecutive tests).^12^ In addition, physical activity information was collected by questionnaires. 

The level of physical activity was assessed using the International Physical Activity Questionnaire(IPAQ). Thisinstrument was validated by Craig etal.^13^ for use in 12countries and by Pardini etal.^14^ for use in Brazil. Participants were divided in four categories: very active, active, irregularly active and sedentary.

An anthropometric evaluation was performed at the Physical Activity Evaluation and Research Laboratory at the School of Physical Education and Sports Sciences at PUCRS. The following measurements were taken: weight and height; bicep, tricep, subscapular, suprailiac, abdominal, thigh, and calf skinfolds; arm, thigh, and calf circumference on the right side of the body; and femur, humerus, and radius diameter. All of the anthropometric data were collected according to the Anthropometric Standardization Reference Manual Guidelines.^15^ Height was measured using a precision stadiometer (Sanny, Kirchnner & Wilhelm, Medizintechnik, Germany) to the nearest 0.1cm. Weight was measured using a digital scale (0 to 150kg) to the nearest 100g (Filizola S/A, São Paulo, Brazil). Body mass index(BMI) was calculated and normalized to a Z-score using the WHO Antroplus software.^16^

**Figure 1:**
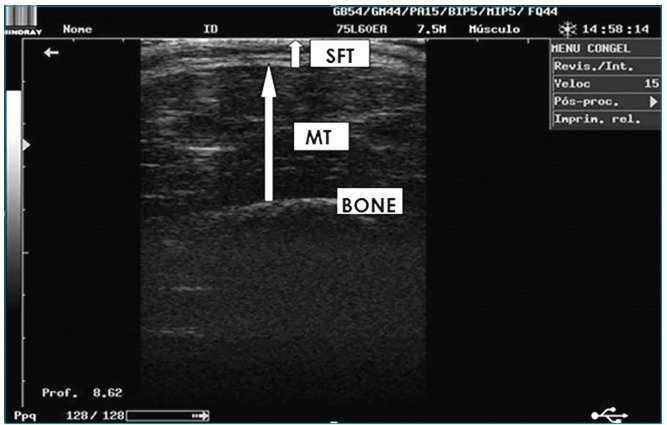
US image of the quadriceps. SFT: subcutaneous fat thickness; MT: muscle thickness.

Ultrasonography was performed to measure muscle thickness and subcutaneous fat. These assessments were performed on the brachial triceps, quadriceps, and medial gastrocnemius on the right side of the body using a DP-6600 Digital Ultrasonic Diagnostic Imaging System (Shenzhen Mindray Bio-medical Electronics Co., China) with a 7.5MHz linear probe. For the scan, patients laid on their backs, keeping the limb to be scanned extended and relaxed. Theequipment was connected to a computer through a USB port. Toobtain B-mode images, ultrasound gel was used and the probe was placed transversely to ensure minimal pressure on the skin. After precise identification of the desired anatomical landmarks, measurements were obtained over the image with the aid of calipers. Subcutaneous fat tissue and bone tissue boundaries were marked. Muscle thickness was defined as the distance between these boundaries.^17^ ^,^ ^18^ Ultrasonographic images of subcutaneous fat were measured between the skin and muscle thickness (Figure1).^17^ ^,^ ^18^ During imaging, subjects remained in a supine position, with the extremity of interest extended and relaxed. Image measurements were performed in the ImageJ software environment. Allimages were performed by the same investigator (RPS), who had been previously trained in the assessment procedures by an experienced investigator (RRB). The trainer also evaluated the quality of the images obtained.

For the skinfold measurement, points were located and marked out in accordance with a specific protocol. For the assessment of body fat in the triceps brachial region, measurements were obtained on the posterior surface of the arm, at the midpoint between the acromion process, andthe lateral epicondyle of the humerus. Toassess body fat in thevastus lateralis region, measurements were obtained in the distal third of the anterior surface of the thigh, which was located by measuring the distance between the greater trochanter and the notch between the tibial and femoral condyles. Toassess body fat in the medial gastrocnemius region, measurements were obtained in the proximal third of the posterior aspect of the leg, which was located by measuring the distance from the notch between the tibial and femoral condyles to the lateral malleolus of the tibia. Body fat percentage was estimated using a previously validated equation, which has been widely used elsewhere in the literature.^15^ ^,^ ^19^ Skinfolds were measured using a Lange skinfold caliper to the nearest 0.5mm (beta Technology Incorporated, Cambridge, Maryland, USA). Also, they were used to determine body fat percentage according to a specific protocol. Body composition was assessed in terms of muscle mass and fat mass. These data were analyzed according to specific equations that have been previously validated.^15^ ^,^ ^19^ ^,^ ^20^

All CF patients had their pulmonary function evaluated using Koko (nSpire Health, Louisville, USA) a flow-based spirometer, following international guidelines. The spirometric parameters evaluated included forced vital capacity (FVC), forced expiratory volume in 1second (FEV_1_) and forced expiratory flow between 25 and 75% of FVC (FEF_25-75%_), and the results were normalized using an international equation.^21^

Sample size was estimated based on the main outcome (muscle thickness). The required sample size for a correlation coefficient of at least 0.45 between US measurements of muscle thickness and the percent of lean mass was calculated as 39participants in each group, considering a loss of 10%. This sample size would be sufficient to detect differences in muscle thickness between CF participants and controls, as described in a previous study^22^ which obtained a standard deviation of 4.7mm for an expected difference of 5mm between groups.

The study protocol was submitted and approved by the University Research Ethics Committee (protocol number 10/05539). Inaddition, all participants or legal guardians agreed to participate in the study and signed the informed consent form. Participants also signed an assent form.

Regarding statistical analysis, the distribution of continuous variables was evaluated using the Kolmogorov-Smirnov test. Variables presenting normal distribution were expressed as mean±standard deviation. Categorical variables were expressed as absolute or relative frequencies. Thecomparison of demographic and anthropometric characteristics and of physical activity and fat data in the two groups was performed using the chi-square test and Student’s ttest for independent samples, depending on the type of variable. Correlations between the variables were evaluated using Pearson’s linear correlation test. Data analyses were carried out using SPSS v. 18.0 (SPSS Inc., USA). Significance was set at p<0.05.

## RESULTS

Thirty-nine children and adolescents with CF and 45 controls were enrolled in the study (61.9% males). Mean age was 13.0±3.4years old and 12.9±3.0 for the CF and control groups, respectively. Nostatistical differences were observed between groups regarding demographic and anthropometric characteristics, except for BMI z-score (p=0.011) and physical activity levels (p=0.002) (Table1). Overall, patients with CF presented low clinical severity status, considering the Shwachman-kulczyki score, as well as a low frequency of chronic colonization by *Pseudomonas aeruginosa*. In addition, Table2 shows a mild pulmonary function (z-score) impairment (FEV_1_: -1.43±2.28; FVC: -1.02±2.04; FEF_25-75%_: -1.62±1.95), as 35.9% of the patients presented a z-score for FEV_1_ less than -2.

**Table 1:** Demographic, anthropometric and clinical characteristics of the participants with cystic fibrosis and controls.

Variables evaluated	Cystic fibrosis (n=39)	Healthy controls (n=45)	p-value
Demographics			
Age, years	13.0±3.4	12.9±3.0	0.875
Sex male, n (%)	22 (56.4)	30 (66.7)	0.334
Anthropometrics			
Height, cm	152.6±18.2	153.9±15.0	0.716
Weight, kg	46.0±17.1	50.6 ±16.1	0.207
BMI, z-score	-0.05±1.2	0.63±1.3	0.011*
Clinical data, n (%)			
Genotype with at least one ΔF508 allele^#^	19 (67.9)	-	-
Pancreatic insufficiency	35 (89.7)	-	-
Chronic *Pseudomonas aeruginosa*	08 (20.5)	-	-
Shwachman-kulczyki score	86.2±12.9	-	-
Physical activity levels, n (%)			
Very active	04 (10.3)	01 (2.2)	0.002*
Active	14 (35.9)	21 (46.7)	
Irregularly active	12 (30.8)	23 (51.1)	
Sedentary	09 (23.1)	zero	

Data expressed as mean and standard deviation or relative and absolute frequency; BMI: body mass index; ^#^Genotype data available for only 28 subjects; *indicates significant differences (p<0.05).

**Table 2:** Pulmonary function data of participants with cystic fibrosis.

Variables evaluated	n=39
Pulmonary function	
FEV_1_, absolute	2.30±1.06
z-score	-1.43±2.28
FVC, absolute	2.83±1.16
z-score	-1.02±2.04
FEV_1_/FVC, absolute	0.79±0.11
z-score	-1.03±1.48
FEF_25-75%_, absolute	2.31±1.33
z-score	-1.62±1.95

Data expressed as mean and standard deviation; FEV1: forced expiratory volume in 1 second; FVC: forced vital capacity; FEF25-75%: forced expiratory flow between 25 and 75% of vital capacity.

Tricep, quadricep and gastrocnemius thickness and arm and thigh circumferences were similar in both groups. However, limb circumference was smaller in CF patients only for the calf (p=0.023). Although most of the diameters (radius and humerus) were similar in both groups, the femur diameter was also smaller (p<0.001) in CF patients as compared to controls. However, there were no significant differences in body composition (muscle mass and fat mass) between the groups (Table3).

**Table 3:** Muscle thickness, limb circumference, bone diameter, body composition, subcutaneous fat and skinfold thickness in participants with cystic fibrosis and controls.

Variables evaluated	Cystic fibrosis (n=39)	Healthy controls (n=45)	p-value
Muscle thickness (cm)			
Triceps	1.4±0.4	1.5±0.4	0.481
Thigh	2.4±0.7	2.5±0.9	0.852
Calf	1.7±0.4	2.0±1.3	0.136
Circumference (cm)			
Arm	23.8±11.9	24.5±4.5	0.713
Thigh	46.5±8.5	50.2±9.6	0.066
Calf	30.2±4.7	32.6±5.0	0.023*
Diameter (cm)			
Radius	4.8±1.6	5.3±0.4	0.080
Humerus	6.3±1.3	6.4±0.8	0.921
Femur	8.0±1.4	9.3±1.1	<0.001*
Body composition			
Muscle mass (kg)	36.0±10.8	36.0±12.4	0.995
Fat mass (%)	22.5±9.4	27.5±14.2	0.061
Subcutaneous fat			
Triceps	0.7±0.2	0.8±0.3	0.247
Quadriceps	0.8±0.3	0.9±0.3	0.439
Gastrocnemius	0.7±0.2	0.7±0.2	0.662
Skinfold thickness			
Triceps	12.9±6.0	16.4±8.4	0.031*
Quadriceps	18.7±6.3	23.5±10.8	0.019*
Gastrocnemius	15.2±6.5	18.5±9.9	0.082

Data expressed as mean and standard deviation. *indicates significant differences (p<0.05).

No significant between-group differences were found when comparing US measurements of subcutaneous fat in the triceps, quadriceps, and gastrocnemius regions. However, a comparison of skinfold measurements revealed significant differences between the two groups, with patients in the CF group exhibiting decreased skinfolds thickness in the triceps (p=0.031) and quadriceps (p=0.019) (Table3).

We observed weak and moderate correlations of quadriceps thickness measured by US with FVC and lean mass, respectively. Likewise, there were moderate correlation coefficients of subcutaneous fat (triceps, quadriceps and gastrocnemius) with BMI, lean mass (only quadriceps) and fat mass in CF patients (Table4). Inaddition, there were correlations between skinfold measurements of triceps (r=0.733; p<0.001), quadriceps (r=0.639; p<0.001), and gastrocnemius (r=0.492; p<0.001) with the BMI.

**Table 4:** Correlation coefficients of muscle thickness and subcutaneous fat measured by ultrasonography including nutritional status and pulmonary function in cystic fibrosis patients.

Variables evaluated	Ultrasonography (muscle thickness)			Ultrasonography (subcutaneous fat)		
Nutritional status	Triceps	Quadriceps	Gastrocnemius	Triceps	Quadriceps	Gastrocnemius
BMI, z-score	0.186	0.182	0.129	0.590**	0.511**	0.475*
Lean mass (%)	0.253	0.424**	0.301	0.206	0.362^*^	0.283
Fat mass (%)	0.144	-0.045	0.048	0.624**	0.594**	0.690**
Pulmonary function, z-score						
FEV_1_	0.021	0.235	0.080	-0.055	-0.117	-0.020
FVC	0.074	0.336*	0.199	-0.012	0.195	0.069
FEF_25-75%_	-0.003	0.092	-0.037	-0.055	-0.117	-0.020

BMI: body mass index; FEV_1_: forced expiratory volume in 1 second; FVC: forced capacity vital; FEF_25-75%_: forced expiratory flow between 25 and 75% of vital capacity. *p<0.05; **p<0.01.

**Figure 2:**
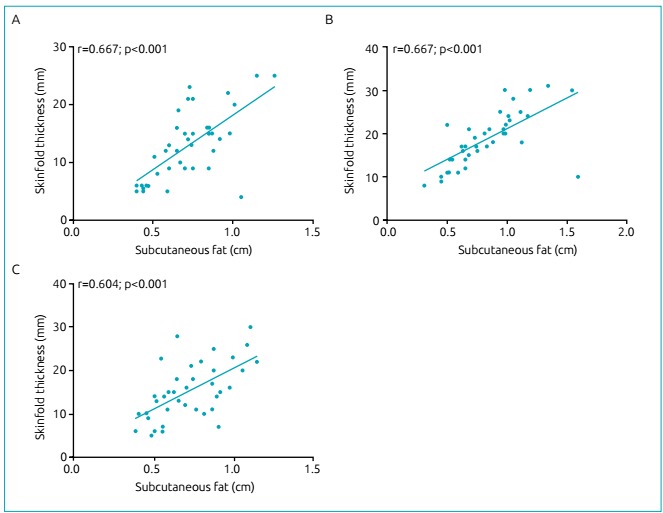
Correlations of the triceps (A), quadriceps (B) and gastrocnemius (C) between US subcutaneous fat and skinfold measurement for the assessment of body fat content in CF patients.

Testing for correlation between US subcutaneous fat and skinfold measurements for an assessment of body fat content revealed significant moderate correlations of the triceps, quadriceps and gastrocnemius in CF patients (Figure 2).

## DISCUSSION

In the present study, US assessment of muscle thickness did not reveal a statistically significant difference between CF participants and healthy controls. Pulmonary function was mildly impaired in CF patients, as reflected by FVC and FEV_1_ z-scores over -1, which corresponded to higher values than 1standard deviation below the mean. We have also reported BMI and body composition findings obtained through anthropometric and skinfold thickness evaluation, as well as subcutaneous US fat measurements. Weidentified lower body fat percentages and significant reductions in body fat in the triceps and quadriceps in patients with CF compared to controls, as measured by skinfold thickness. BMI was within normal limits. Thesefindings reflect a general good nutritional and functional status of the studied subjects.

Nutritional status is strongly linked to pulmonary impairment in CF in the long term. Weight loss and undernourishment resulting from the co-occurrence of increased energy expenditure and decreased energy intake caused by anorexia lead to a decrease in lean mass and muscle mass, including respiratory muscle mass.^23^ Themaintenance of lean mass is involved in the preservation of pulmonary function in CF patients. Conversely, loss of lean mass has been associated with overall disease severity, decreased pulmonary function, respiratory muscle weakness, and increased systemic inflammatory activity.^6^ ^,^ ^24^

Analyses of individual body composition components revealed that fat and bone masses were lower in CF participants. Muscle mass or muscle thickness, however, were not different between the groups. Inthe presence of weight loss, fat is usually among the first components to be depleted. Because nutritional status was not significantly compromised in this group of CF patients, a difference in muscle mass was not detected. Different body composition components influence limb circumference measurements. When comparing with healthy subjects, CF patients had lower fat and bone components and consequently smaller limb circumferences, although muscle mass and muscle thickness were not statistically different. The inclusion of patients with more severe CF might have produced different results.^25^ Also, bone mass estimates were smaller in CF patients. Bonesize is related to calcium intake and physical activity. In addition, malabsorption, which is commonly found in this population, may at least partially explain this finding.^26^

Several methods are available for an assessment of body composition, and they differ in terms of physical basis, cost, accuracy, ease of use, and ease of equipment transport. Anthropometry has been reported as the preferred parameter for assessing nutritional status in collective settings,^27^ particularly in childhood and adolescence, due to its ease of implementation, low cost, and harmless nature.^28^ Although BMI is currently one of the anthropometric parameters most widely used for population-wide evaluations of nutritional status in epidemiological studies,^27^ ^,^ ^29^ its use has many limitations, as it cannot provide information on body composition or body fat distribution.^29^ Skinfold measurements have also been a widely used method for body fat quantification, in view of its low operating costs and relative simplicity in relation to other techniques.^30^ Inthis study, we evaluated US as an alternative method for estimating subcutaneous fat content. Ultrasounds are a safe, noninvasive method associated with minimal discomfort and relatively low cost for the quantification of muscle and adipose tissue. Their use for clinical assessments at bedsides and in outpatient settings is becoming increasingly widespread.^7^ These features give US the potential for particular utility in the assessment of young children, who are relatively intolerant to being handled, in addition to bedbound patients.

In the present study, we found significant, moderate, and positive correlations between skinfold measurements and US findings of body fat content in the triceps, thigh, and calf regions, which suggests a potential role for ultrasounds in the assessment of this nutritional parameter. Asin the present study, Neves etal.,^31^ evaluating a sample of 195male soldiers, found significant correlations between estimates of subcutaneous fat content obtained by a portable ultrasound and by a skinfold thickness evaluation at all measurement points. Correlations were greater in the thigh (0.715) and triceps (0.547) than in the calf region (0.249).

Although muscle thickness was not different between the groups, the positive correlation observed between quadriceps and FVC indicates a trend toward worse pulmonary function in patients with less muscle mass. FVC, among other factors, depends on chest muscles, which influence maximal inspiration to total lung capacity and maximal expiration. Onthe other hand, FEV_1_ is more significantly influenced by bronchial obstruction, with a closer link to pulmonary flows rather than to lung volumes. CF patients have bronchial obstruction that is secondary to their genetically inherited pathophysiologic defect together with recurring inflammation and infections, even at initial stages of the disease, in which muscle depletion may not yet be present.^32^ Since the correlation with spirometry was only observed for the thigh but not for other muscles, the quadriceps may be a potential marker of CF functional impairment.

The present study has limitations that must be addressed. Firstof all, no reference values are available for muscle thickness in children and adolescents. Wetried to overcome this limitation by including a control group with similar age and sex distribution. Also, although ultrasound measurements were calculated using machine software, the operator was not blinded to the patient’s group. Another limitation is that nutritional and pulmonary impairment were only mild in our CF group. Moreconclusive results might have been obtained had we studied adult individuals, with more advanced lung disease. In addition, the utility of this method for nutritional assessment requires further validation before it can be recommended for routine use.

In summary, we observed a reduction in subcutaneous fat content in this population of patients with CF. Quadricep muscle thickness correlated with FVC and nutritional parameters, as well as subcutaneous fat correlated with skinfold measurements, indicating that US may be an alternative tool for the assessment of body composition. However, additional studies with larger populations affected by more severe diseases are required to confirm and further elucidate these findings.
